# Forensic postmortem findings for sudden unexplained death in schizophrenia: case series and literature review

**DOI:** 10.3389/fpsyt.2026.1578123

**Published:** 2026-01-29

**Authors:** Yuanyuan Chen, Fengping Yan

**Affiliations:** Department of Forensic Medicine, School of Basic Medical Sciences, Gannan Medical University, Ganzhou, Jiangxi, China

**Keywords:** antipsychotics, autopsy, forensic pathology, schizophrenia, sudden unexplained death

## Abstract

**Introduction:**

There are currently limited autopsy-based studies on sudden unexplained death in patients with schizophrenia (SDU-SCZ).

**Methods:**

We summarized the demographic data, autopsy characteristics, and postmortem antipsychotics result for a total of 152 SUD-SCZ decedents, encompassing three cases from our forensic center and 149 literature-reported autopsy cases.

**Results:**

The SUD individuals were found in adults at all ages, ranging from 19–86 years old, with a male-to-female ratio being 94: 58. A total of 106 patients (69.7%, 106/152) were documented to be overweight or obese. Autopsy findings were available in 77 of the 152 cases. The most frequent postmortem pathology was cardiac (46.8%, 36/77), of which unclassified cardiomegaly, focal myocardial fibrosis, and mild coronary atherosclerosis were the most common manifestations, documented in 11 (14.3%), 8 (10.4%), and 5 cases (6.5%), respectively. Data on postmortem antipsychotics were available in 74 of the 152 cases, of which 65 (87.8%, 65/74) were tested positive of any antipsychotic drug, all at therapeutic levels. Olanzapine and clozapine were the most commonly prescribed antipsychotic drugs, documented in 18 cases (24.3%, 18/74) and 16 cases (21.6%, 16/74), respectively. In these SUD-SCZ individuals, the exact cause of death remained unexplained after comprehensive autopsy examination and postmortem antipsychotics analysis.

**Discussion:**

Linking premorbid conditions (e.g. overweight or obese) to antipsychotics medication histories and postmortem myocardial pathologies would facilitate a more accurate determination and interpretation of the cause of death. Forensic investigation is useful for developing preventive strategies for this vulnerable population.

## 1 Introduction

Schizophrenia is a severe mental illness that affects approximately 1% of the world’s population. Life expectancy in schizophrenia patients is estimated to be 10 to 25 years less than the general population and the incidence of sudden death is about three to four times higher (1, 2). Sudden cardiac death accounted for up to 50% of all deaths in schizophrenia, with the primary cause of death due to structural heart disorders, including coronary heart disease, cardiomyopathies, and myocarditis. Despite those with identifiable abnormalities, some cases, about 8 to 10%, may have no definitive cause of death after systemic autopsy and toxicological screening. The causes of death remained unexplained, and were mostly presumed to be due to fatal arrhythmias (3, 4). In forensic pathology, sudden deaths that remain unexplained after comprehensive pathological and toxicological examinations were categorized as sudden unexplained death (SUD) (5). Those sudden deaths in schizophrenia patient without identifiable cardiac abnormality were commonly referred as sudden unexplained death in schizophrenia (SUD-SCZ) (6).

Most recent studies continue to highlight the elevated risk of sudden cardiac death and premature mortality in schizophrenia (7–9). Specifically, a large-scale systematic review and meta-analysis of 135 retrospective, nationwide, and targeted cohort studies assessing mortality risk among people with schizophrenia compared to the general population or other controls between 1957–2021 established a 152% increased risk in all-cause mortality, with a 100-200% risk for cardio-cerebrovascular or any natural causes of death (10). Despite the acknowledged increased of all-cause and cause-specific mortality rate observed in patients with schizophrenia as compared to the general population, fatal cardiac arrhythmias (absence of typical cardiac pathology) as the cause of sudden death among patients with schizophrenia were less frequently noted in the literature (11). Risk factors and the underlying mechanisms for SUD-SCZ remain unclear. The relevant autopsy-based studies on this topic are also scarce. In this study, we presented three cases of SUD-SCZ autopsied at Forensic Center of Gannan Medical University and further conducted a literature search in Pubmed online database for autopsy studies comprising SUD-SCZ cases. We sought to provide a comprehensive summary and novel insights into SUD-SCZ from a forensic perspective.

## 2 Case presentation

SUD-SCZ cases were retrospectively identified from autopsies that were performed at Forensic Center of Gannan Medical University between January 2020 and December 2024. Cases were identified to be included if the decedents were clearly diagnosed of schizophrenia in clinic before death, experienced a sudden, unexpected and natural death, and were absence of definite anatomic or toxicological cause of death after comprehensive postmortem examination. Postmortem examination consists of medical history review, scene investigation, autopsy, and antipsychotics analysis in blood. Macroscopic and microscopic evaluations of the hearts were performed by forensic pathologists with expertise in cardiovascular pathology. Cases with the cause of death being suicide, homicide, and drug overdose, or with significant heart diseases identified at autopsy, were excluded.

Three cases were identified. Medical information and forensic findings of the three decedents were extracted from archived records. Data were analyzed anonymously. Review of the records was approved by the Ethical Review Board at the School of Basic Medical Science, Gannan Medical University (Approval No.: 2021-127, 2023-051).

### 2.1 Case 1

**Figure 1:**
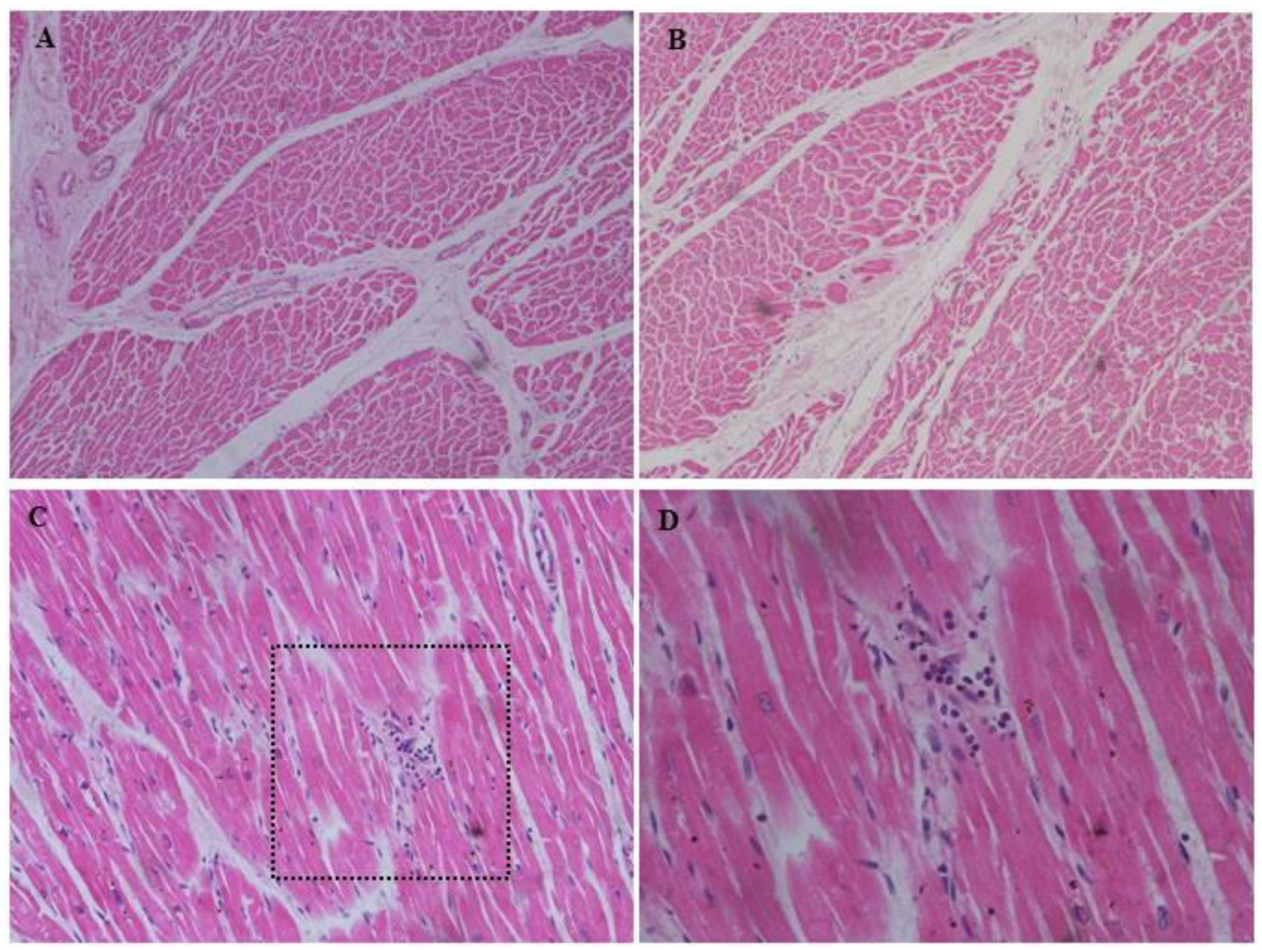
Cardiac histo-pathological findings of perivascular fibrosis **(A)** and focal myocardial fibrosis in left ventricle **(B)** in case 1; lymphocytic infiltrate within the myocardium in the absence of significant myocyte injury **(C)** with detailed enlargement **(D)** in case 3. Magnification: **(A, B)** 100×, **(C)** 200×, **(D)** 400×. All with H&E staining.

A 44-year-old man was found dead in a psychiatric hospital. He was diagnosed with schizophrenia for over 10 years. He routinely received antipsychotic drug treatments, including clozapine, risperidone, and olanzapine, but with a poor compliance. He was a heavy drinker and smoker. He was admitted to a psychiatric hospital due to aggressive psychotic behavior. During hospitalization, he took olanzapine 10mg/day orally for the first 4 day, followed by risperidone 4mg/day orally for the next 8 days, with diazepam 10mg/day. In the 14th day, he suddenly became unresponsive, stopped breathing, and was soon announced death in the hospital. At autopsy, the heart weighted 315g with no coronary atherosclerosis. Perivascular fibrosis and myocardial fibrosis in left ventricle were observed microscopically (**Figures 1A, B**). The liver presented with mild fatty changes microscopically. Other organs were unremarkable. Olanzapine and risperidone was positive in blood via postmortem antipsychotics screening.

### 2.2 Case 2

A 47-year-old woman collapsed with loss of consciousness, and died immediately in a psychiatric hospital. She was diagnosed with schizophrenia for two years. She was given several different antipsychotic drugs including quetiapine, olanzapine, but withdrawn when symptoms being improved. She was diagnosed with diabetes. After becoming irritability with odd behaviors for 3 days, she was referred to a psychiatry hospital. During hospitalization, risperidone 2mg/day was orally prescribed. The medication lasted for 2 days until her sudden death. At autopsy, the heart weighted 260g with no coronary atherosclerosis. The liver presented with mild fatty changes microscopically. Other organs were unremarkable. Risperidone was positive in blood via postmortem antipsychotics screening.

### 2.3 Case 3

A 48-year-old woman was found dead in an early morning at her residence. She was a chronic schizophrenia since age 36 and was treated several times. Antipsychotic drugs included risperidone, olanzapine. She was diagnosed with diabetes and hyperlipidemia, but whether she was on the treatment of diabetes and how was her blood glucose controlled remained unknown. Her last therapy lasted for a total of 14 days four months prior to her death. She took olanzapine 15mg/day orally for eight days, followed by amisulpride tablets 600mg/day orally for the next six days. Lorazepam 0.5mg/day was also prescribed. At autopsy, the heart weighted 220g. Small areas of focal inflammatory infiltration were observed in the myocardium, without significant myocyte injury microscopically (**Figures 1C, D**). No significant abnormality was found in other organs. Olanzapine and amisulpride were identified in blood via postmortem antipsychotics screening.

## 3 Literature review

**Figure 2:**
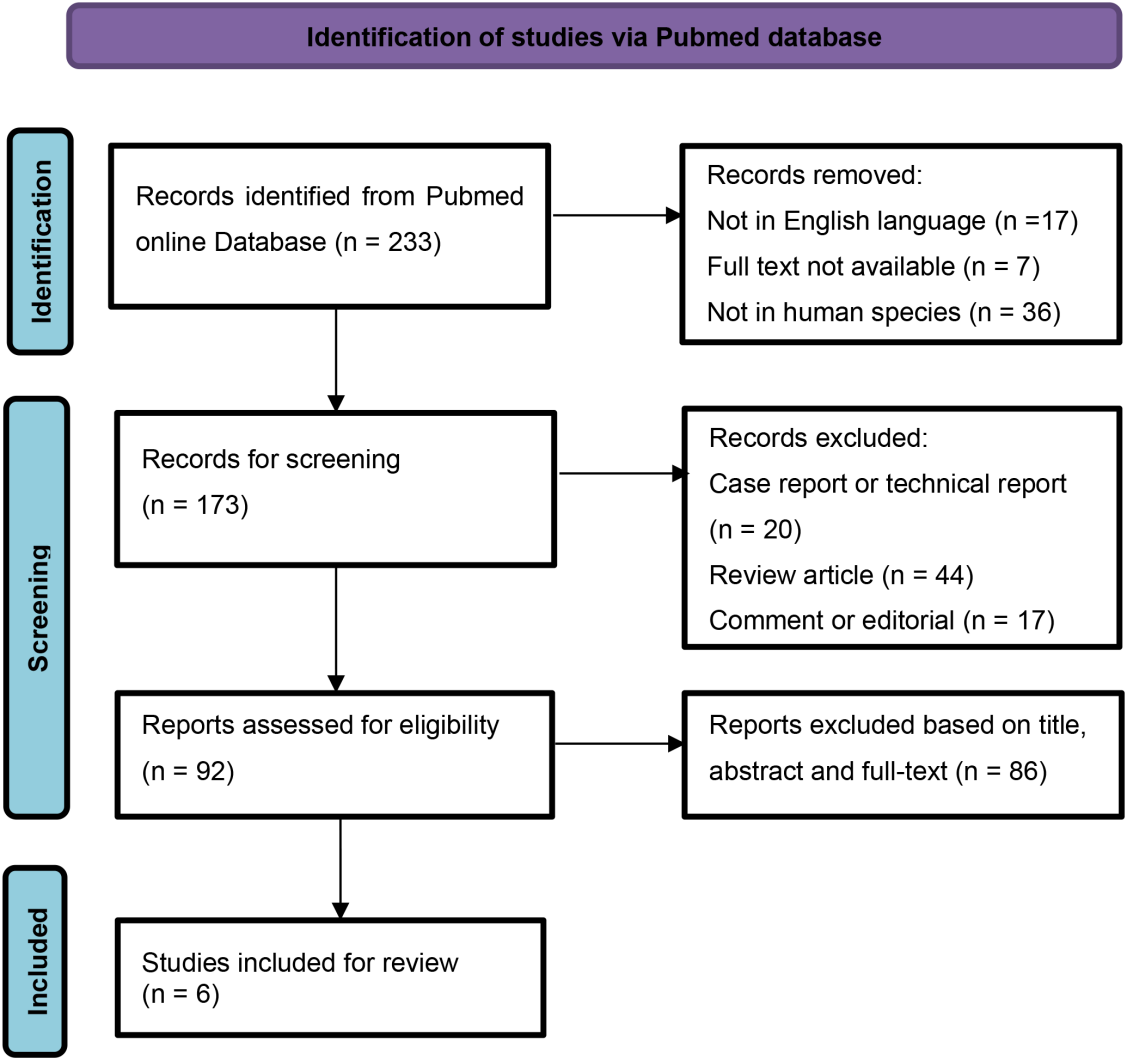
PRISMA flow chart for study selection.

We conducted a literature search in Pubmed online database for forensic studies comprising SUD-SCZ cases, in accordance with the 2020 Preferred Reporting Items for Systematic Reviews and Meta-Analyses (PRISMA) statement guidelines (12). Two authors, Chen and Yan performed online search independently by utilizing the keywords “sudden death” and “schizophrenia” to detect all publications that related to autopsies in SUD-SCZ. The search was limited to articles published in English language. Restriction in publication date was set from January 2000 to July 2025. Articles were included in if they were forensic studies comprising SUD-SCZ cases, with full text available. Reviews, technical reports, case reports, books, or any published material without complete information were excluded. Titles, abstracts and full text were carefully evaluated to select eligible literature. In cases of discrepancy, two authors reached a consensus through discussion. A manual search of references was performed in the included articles to identify additional studies. PRISMA flowchart summarizing the article selection procession is provided in **Figure 2**.

Six forensic studies containing 149 SUD-SCZ cases were identified through the literature search (13–18). With the additional three from our own case series, a total of 152 cases were included in this study. Basic information and forensic characteristics of the 152 SUD decedents were summarized in **Table 1**.

**Table 1:** Basic information and forensic characteristics of SUD-SCZ individuals (n=152).

Category	Rosh A, et al (13)	Sweeting J, et al (14)	Ifteni P, et al. (15)	Sun D, et al. (16)	Wang S, et al. (17)	Vohra J, et al (18)	Present
Publication year	2003	2013	2014	2019	2023	2025	
Country	USA	Australia	Romania	USA	China	Australia	China
Data range (y)	1993-2001	2003-2012	1989-2013	2008-2012	2010-2022	2016-2021	2020-2024
Autopsies in SCZ (n)	216	683	51	391	NA	NA	NA
SUD-SCZ Cases (n)	6	72	6	11	18	36	3
Age range (years)	35-54	22-86	32-66	27-66	19-53	18-65	44-48
BMI (kg/m^2^)	NA	26.0 ± 7.1	26.0 ± 4.8	31.0 ± 7.2	NA	(a)	NA
Male/Female	4/2	41/31	4/2	7/4	11/7	26/10	1/2
Autopsy findings (b)		NA					
Cardiomegaly				1		10	
Ventricle dilation	2			2			
Mild atherosclerosis	1		3		1		
Focal myocardial fibrosis				2	1	4	1
Focal myocardial inflammation						3	1
Other cardiac pathology			2		2		
Pulmonary congestion	2						
Focal bronchopneumonia					3		
Hepatocyte steatosis	1				7		2
Postmortem antipsychotics (c)		NA	NA				
Chlopromazine	1				5		
Haloperidol	1				2		
Benztropine	2						
Thioridazine	1						
Clozapine	1				5	10	
Olanzapine	1				5	10	2
Quetiapine				4	1		
Risperidone				2	5		2
Ziprasidon				1			

SUD, sudden unexplained death; SUD-SCZ, sudden unexplained death in patients with schizophrenia; BMI: body mass index; NA: not available; Other cardiac pathology: including myocardial dystrophy, chronic pericarditis, and cardiac fatty infiltration; (a) 17/36 of the SUD subjects were obese with BMI>30; (b) autopsy findings were available in 77 cases; (c) postmortem antipsychotics data were available in 74 cases, of which 65 were tested positive, 29 cases were recorded as poly-pharmacy.

These unexplained deaths were found in adults at all ages, ranging from 19–86 years old, with a male-to-female ratio being 94: 58. The decedents were reported to be relatively young in two of the publications. Nine of the 11 patients were younger than 50 years of age with an average age of 38 years (16). The average age of the SUD group (39.0 ± 8.4 years) was reported to be significant younger compared to the sudden cardiac death group (49.5 ± 13.0) (17). In three of the included articles, the authors described that all the SUD decedents were overweight or even worse (BMI>25) (14–16). In another study, almost half (17/36) of the SUD subjects were obese with BMI>30, nearly a third (13/36) of the decedents had a premorbid diagnosis of either dyslipidemia (n=6), diabetes (n=4), or hypertension (n=3) (18). A total of 106 patients (69.7%, 106/152) were documented to be overweight or obese (BMI>25).

Autopsy findings were available in 77 of the included 152 cases. The most frequent postmortem pathology was cardiac (46.8%, 36/77), followed by hepatic (13%, 10/77) and pulmonary (6.5%, 5/77). Unclassified cardiomegaly, focal myocardial fibrosis, and mild coronary atherosclerosis were the most common cardiac pathologies, documented in 11 (14.3%), 8 (10.4%), and 5 cases (6.5%), respectively. Hepatic steatosis was documented in 10 cases. Pulmonary congestion or focal broncho-pneumonia were each documented in 5 decedents.

Data on postmortem antipsychotics were available in 74 individuals of the included 152 cases, of which 65 (87.8%, 65/74) were tested positive, all at therapeutic levels. Of the 65 cases, 29 (44.6%, 29/65) were recorded as poly-pharmacy. Olanzapine and clozapine were the most common antipsychotic drugs, recorded in 18 and 16 cases, respectively.

The causes of death were mostly presumed to be due to antipsychotics associated cardiac arrhythmia (13, 14, 16–18). However, Ifteni et al. (15) found no evidence to support the antipsychotic-induced arrhythmias as cause of death, as the utilization of antipsychotic drugs was similar in the explained and unexplained death groups.

## 4 Discussion

Patients with schizophrenia were at a high risk of sudden cardiac death, with the primary cause of death due to structural heart disorders, including coronary heart disease, myocarditis, and cardio-myopathy. In some of these patients, the exact cause of death remained unexplained after standardized autopsy examination procedures, and these cases were categorized as SUD-SCZ. SUD accounted for a small minority of excess deaths in people with schizophrenia with a reported incidence ranging from 2.8% (13) to 11.8% (16). A recent retrospective study from Australia indicated that the prevalence of SUD-SCZ was up to 29.2% among patients with schizophrenia (19). Our cases, with comprehensive postmortem examination, add to the very few autopsy studies in this population published in the literature. Further research is required to explore the increased observation of SUD in people with schizophrenia.

According to our reported and literature-derived cases, the SUD individuals were found in adults at all ages, ranging from 19–86 years old, with the male-to-female ratio being 94: 58. Two out of the six studies reported that young individuals accounted for the majority of the decedents (16, 17), which raises the possibility that male sex or young age might be a risk factor for SUD-SCZ. However, a meta-analysis of 43 studies reporting on 2,700,825 people with schizophrenia demonstrated that both males and females with schizophrenia experience increased risks of all-cause and cause-specific mortality compared to the general population. There was no statistical significant difference in sex-dependent mortality risk except for males being at a significantly higher risk of death due to dementia (20). The differential influence of sex and age on SUD-SCZ merits further investigations.

Some risk factors have been proposed for SUD in patients with schizophrenia and other psychiatry disorders. An earlier study comprising 100 SUD patients relying on death certificates indicated that dyslipidemia (p=0.012) and co-morbid dyslipidemia and diabetes (p=0.008) were more common in the unexplained cases compared with the group of explained sudden deaths in psychiatric patients (21). In the present study, among the retrieved articles, three articles described that all the SUD decedents were overweight or even worse (BMI>25) (14–16), whereas another study noted that almost half (17/36) of the SUD subjects were obese with BMI>30, summarizing that nearly a third (13/36) of the decedents had a premorbid diagnosis of either dyslipidemia, diabetes, or hypertension (18). In our three cases, two had diabetes and one had dyslipidemia. Our data confirms the notion that dyslipidemia and diabetes were common in SUD patients with schizophrenia. These risk factors may at least to some extent, either alone or in combination, correlate with a more rapid illness worsening than in patients without mental illness (22). The rate of patient with severe mental illness not treated for established cardiovascular risk factors has been reported to be as 88% for dyslipidemia, 62% for hypertension, and 30% for diabetes (23, 24). It is hence strongly suggested that clinicians remain vigilant for the signs or symptoms of diabetes, or dyslipidemia to improve cardiovascular safely in this vulnerable population. A total of 106 patients (69.7%, 106/152) were documented to be overweight or obese (BMI>25) in our study, highlighting the importance of tailored metabolic interventions based on BMI stratification. It is noteworthy that a significant higher occurrence of psychiatric episodes was observed in the SUD group than in explainable deaths (p=0.018) and (OR = 4.025, P = 0.040) (17). Psychiatric episodes were considered as an independent risk factor for SUD in schizophrenia (17). Persistent psychotic episodes often demand an extra load on the heart, and were also associated with increased risks of short-term post-acute coronary syndrome, thereby increasing the possibility of sudden death in patient with schizophrenia (25). Risk factors for sudden death in schizophrenia are complex. Patients with schizophrenia and other psychiatric disorders are more likely to be overweight, smoke, and have diabetes, hypertension, dyslipidemia, or metabolic disorders than those without schizophrenia (26, 27) Moreover, long-term treatment with antipsychotic medication can increase the risk of diabetes, hypertension and hyperlipidemia, even in young patients. This state of metabolic change may lead to an increased risk of sudden cardiac death and cardiovascular disease mortality among schizophrenia (28, 29). Aside from the aforementioned cardiovascular risk factors, other factors, such as drug influences, genetic background might also underlie predisposition to sudden death (30). Awareness of these risk factors and implementing proactive management might lead to effective interventions for prevention and treatment in schizophrenia patients.

At autopsy, although no clear causes of death were determined, some pathological changes were still observed in our reported and those literature-derived cases, with the cardiac alterations being particularly prominent (46.8%). Unclassified cardiomegaly, focal myocardial fibrosis, and mild coronary atherosclerosis were the most common cardiac pathologies, followed by focal myocardial inflammation, myocardial dystrophy, and cardiac fatty infiltration. These cardiac pathologies, were insufficient to meet diagnostic criteria for known pathologies and insufficient to conclude as causes for sudden death. Such alterations may be partly due to the effects of antipsychotic medications on the cardiac tissue, as chronic exposure to antipsychotics have been reported to impair cardiac structure and function, causing inflammatory lesions, cardiac fibrosis, and cardiomyopathy (31, 32). Taking the focal myocardial inflammation and focal myocardial fibrosis mentioned in our cases for example, clozapine induced myocarditis is by far the most frequently reported antipsychotic-related cardiac injury, with an estimate incidence of 0.7-1.2% (32, 33). Most of these cases occurred in the first 2 months after clozapine therapy. If acute myocarditis is not recognized at the early stage, it may progress to dilated cardiomyopathy, characterized by ventricular dilation and heart dysfunction. Many of the patients were entirely asymptomatic and further, did not undergo the expected investigations that one might expect prior to death (34, 35). In an autopsy report, six in 14 cases were reported to die suddenly from dilated cardiomyopathy, following chronic antipsychotic use (36). A recent study by Esposito et al. from Italy identified myocardial fibrosis and contraction band necrosis in young schizophrenia patients without pre-existing cardiac disease, died suddenly while on antipsychotic therapy at therapeutic doses, corroborating the findings from our cases (37). However, such cardiac findings have also been observed occasionally by forensic pathologists in otherwise healthy individuals, or in SUD among general population, and were categorized as non-diagnostic or autopsy findings with uncertainty of significance (38, 39). From a forensic point of view, exploration of the comparative autopsy findings on preponderance and severity in patients with antipsychotics exposure and those non-exposed population is important for cause-of-death determination. In addition, a pragmatic close clinical monitoring protocol including cardiac biomarkers aimed at timely detection of cardiac toxicity (cardiac injury), in the initial phase of antipsychotics treatment is strongly recommended.

The exact pathophysiological causes of SUD-SCD are not fully understood yet. It is very probable that arrhythmias potentially related with antipsychotics use may play a significant role (40, 41). Antipsychotic medications may increase the risk of serious ventricular arrhythmias such as TdP, probably QT interval prolongation through blockade of potassium ion channels, which in worst case, can lead to sudden cardiac death. Specifically, poly-pharmacy and high-dose antipsychotics are widely considered to be an important and the clearest evidence for an elevated SCD risk related to drug-drug interactions in pharmacodynamics and pharmacokinetics (41–44). It is not surprisingly that the majority of the patients in present study were tested positive for antipsychotics, because these patients often receive antipsychotic treatments. However, due to the heterogeneity of antipsychotics medication histories, current available data do not allow to document any clear-cut differences in the risk of sudden death between first and second generation antipsychotics or between individual antipsychotic drugs. Moreover, the aforementioned data do not allow to establish a causal correlation between antipsychotics and fatal cardiac arrhythmia because the absence of clinical electrocardiographic (ECG) evidence. The causes of death were made by excluding other alternative causes of death in the literature and our case series. Linking ante-mortem ECG to antipsychotics medication histories and postmortem myocardial lesions would improve the precise diagnosis (45). In a recent study by LC-MS/MS analysis in antipsychotics treated mice, the authors found that the serum of olanzapine and clozapine were generally within therapeutic ranges, while the olanzapine and clozapine concentrations in the heart were significantly elevated since the day 14 and its average level increased by up to 3-fold and 22-fold on day 21 respectively, suggesting that antipsychotics accumulated in the heart and exclusively induced cardiac toxic effects. This may, on one hand, partially explains the contribution of antipsychotic drug use to the development of SUD, and on the other hand suggests that the antipsychotic drug doses in the heart, not only in blood, should be analyzed for interpretation of unexpected death (46). From a forensic perspective, quantifying drug levels within myocardium and comparing distribution across regions (sub-endocardium, conduction system) would help distinguish direct myocardial accumulation from peripheral redistribution and support mechanistic links to the sudden death (45, 47).

Our study has some limitations. Firstly, the sample size from our center is small, providing a lack of representativeness. Secondly, our literature search used only one database, with the included articles only in English language. Therefore, there may be a portion of literatures being missed. Lastly, neuro-pathological changes were not mentioned or not available in many cases of our study. So, our study did not summarize the neural pathology.

## 5 Conclusions

In all, we systemically analyzed the forensic findings including demographic data, autopsy characteristics, and postmortem antipsychotics results from both our forensic center and literature resources. In these SUD-SCZ individuals, the majority of the decedents were found to be overweight or obese; cardiac pathology was the most common finding at autopsy; olanzapine and clozapine were the most frequently prescribed antipsychotic drugs; the exact cause of death remained unexplained after comprehensive autopsy examination and postmortem antipsychotic analysis. Linking premorbid conditions (e.g. overweight or obese) to antipsychotics medication histories and postmortem myocardial pathologies would facilitate a more accurate determination and interpretation of the cause of death. Forensic investigation is useful for developing preventive strategies for this vulnerable population.

## Data Availability

The original contributions presented in the study are included in the article/supplementary material. Further inquiries can be directed to the corresponding author.
